# An Overview of Rheumatoid Arthritis-Associated Dry Eye Disease, Scleritis, and Peripheral Ulcerative Keratitis

**DOI:** 10.3390/jcm15093207

**Published:** 2026-04-23

**Authors:** María García Forestier, Ricardo Murati Calderón, Armando Oliver

**Affiliations:** Department of Ophthalmology, School of Medicine, University of Puerto Rico, San Juan 00936-5067, Puerto Rico

**Keywords:** rheumatoid arthritis, ocular manifestations, keratoconjunctivitis sicca, scleritis, peripheral ulcerative keratitis, immunosuppression, biologic therapy, JAK inhibitors

## Abstract

Rheumatoid arthritis (RA) is a systemic autoimmune disease that can involve the ocular surface and deeper ocular tissues, leading to a spectrum of ophthalmic manifestations ranging from dry eye disease to vision-threatening inflammation, such as scleritis and peripheral ulcerative keratitis (PUK). This paper presents the results of a narrative review conducted using PubMed and Google Scholar from database inception to March 2026. Eligible publications describing clinical features and management of RA-associated ocular disease were synthesized, and no unpublished data were included. According to the literature, dry eye disease (DED) is the most frequent ocular manifestation of RA, and it is primarily managed with lubrication and topical anti-inflammatory therapies, including cyclosporine and lifitegrast. Additional options for refractory disease include neurostimulation and evaporation-targeted therapy. Scleritis and PUK are less common but represent severe inflammatory complications that generally require systemic immunosuppression. Conventional management includes systemic corticosteroids and steroid-sparing agents such as methotrexate (MTX), azathioprine (AZA), cyclophosphamide (CYC), and mycophenolate mofetil (MMF) in aggressive cases. Escalation to biologic disease-modifying antirheumatic drugs (bDMARDs), specifically tumor necrosis factor-alpha (TNF-α) inhibitors and rituximab (RTX), is supported for refractory scleritis and corneal melt, although evidence is largely observational. Among anti-TNF agents, monoclonal antibodies, such as infliximab and adalimumab, appear more effective than etanercept for ocular inflammation. Rituximab is preferred for vasculitis-associated or refractory disease, and Janus Kinase (JAK) inhibitors represent an emerging option requiring careful safety monitoring. Evidence for DED therapies includes randomized controlled trials (RCTs), whereas data for RA-associated scleritis and PUK are largely derived from registries, case series, and case reports. Prospective studies with standardized ocular outcomes are needed to refine treatment algorithms and compare the effectiveness of biologic versus targeted synthetic agents.

## 1. Introduction

Rheumatoid arthritis is a chronic inflammatory autoimmune disease that primarily affects the synovial joints. Beyond joint destruction, RA has a significant extra-articular presentation, with 40% of patients experiencing systemic manifestations [1,2,3,4]. Ocular involvement is among the most frequent extra-articular manifestations, occurring in roughly 18–28% of RA patients [1,4,5]. In some cases, the eye can be the initial site of presentation [1]. The spectrum of eye diseases associated with RA includes keratoconjunctivitis sicca (KCS; dry eye disease), episcleritis, scleritis, peripheral ulcerative keratitis, and, less commonly, posterior segment involvement, such as retinal vasculitis [6].

While Sjögren syndrome has higher rates of dry eye disease (89%), it is also common in RA (15–28%) [4]. RA is also the most common underlying cause of necrotizing scleritis and PUK, two of its most serious sight-threatening ocular complications [7]. Patients who are positive for rheumatoid factor (RF) and anti-citrullinated protein antibody (ACPA) have a higher likelihood of eye involvement [2,8]. Ocular manifestations often indicate active systemic inflammation or vasculitis and may signal worse prognosis [2,8]. For example, necrotizing scleritis or PUK in RA has historically been associated with increased mortality due to systemic vasculitic complications [9]. Thus, promptly recognizing and treating ocular RA manifestations is essential to preserve vision and reduce systemic morbidity [1,5].

This review provides a comprehensive analysis of RA’s most common ophthalmic manifestations, with a focus on dry eye, scleritis, and PUK, and summarizes both traditional management and recent advances in therapy. We emphasize the importance of a multidisciplinary approach in treating RA and its manifestations to prevent sight-threatening complications.

## 2. Materials and Methods

### 2.1. Search Strategy and Data Sources

We conducted a narrative review of RA-associated dry eye disease, scleritis, and PUK using PubMed (National Library of Medicine, Bethesda, MD, USA) and Google Scholar (Google LLC, Mountain View, CA, USA) from database inception to March 2026. The search was limited to English and Spanish human studies. Search terms included combinations of “rheumatoid arthritis” AND (“dry eye” OR “keratoconjunctivitis sicca” OR “scleritis” OR “peripheral ulcerative keratitis” OR “corneal melt”) AND (“treatment” OR “therapy” OR “biologic” OR “rituximab” OR “TNF” OR “JAK inhibitor”). Reference lists of relevant articles and recent reviews were hand-searched to identify additional publications.

### 2.2. Eligibility Criteria and Study Selection

We included randomized controlled trials, cohort studies, case–control studies, registry analyses, systematic reviews/meta-analyses, case series, and case reports describing clinical features and/or management of RA-associated ocular disease. Titles and abstracts were screened for relevance, followed by full-text review of eligible articles.

### 2.3. Data Extraction and Evidence Appraisal

Study identification, screening, and eligibility assessment were performed by M.G.F., and a subset of articles was cross-checked by R.M.C to ensure consistency and reduce selection bias. Reference management and organization were performed using EndNote X20 (Clarivate, Philadelphia, PA, USA). For each article, we extracted ocular phenotype, study design, sample size, systemic disease context, interventions, and reported outcomes. Because most evidence for severe ocular RA is derived from observational studies and small case series, findings were synthesized narratively and limitations were emphasized. No meta-analysis was performed.

## 3. Dry Eye Disease

### 3.1. Overview

Dry eye disease, or keratoconjunctivitis sicca, is the most common ocular presentation in RA, reported in approximately 15–28% of patients [2,4]. RA-associated dry eye often results from autoimmune-mediated damage to the lacrimal glands and the ocular surface [10,11]. The mechanism involves T-cell infiltration of lacrimal glands, circulating antibodies against glandular receptors, and local release of proinflammatory cytokines causing neurosecretory blockade [11]. In some patients, associated Sjögren syndrome coexists, characterized by autoimmune lymphocytic infiltration of exocrine glands and marked aqueous tear deficiency [11,12].

RA-associated dry eye is often multifactorial, with contributions from both aqueous-deficient and evaporative mechanisms. In addition to lacrimal gland dysfunction (aqueous deficiency), meibomian gland dysfunction (MGD)—dysfunction of the eyelid oil glands that produce meibum, which is crucial for a healthy tear film—can destabilize the tear film and increase evaporation. As such, patients may have a mixed phenotype that influences testing results and treatment selection [11,13].

Notably, simple KCS in RA is usually not correlated with joint disease activity [1,14], although one study found that meibomian gland dysfunction was associated with higher RA disease activity scores (*p* < 0.05) [13]. Patients typically experience dryness, grittiness, redness, foreign body sensation, and fluctuating blurred vision. On examination, it can manifest in reduced tear meniscus, a positive Schirmer test in the case of low tear production, and punctate corneal staining [2,11]. While mild dry eye causes irritation, severe cases can progress to chronic keratitis, corneal epitheliopathy, or corneal ulceration, which are vision-threatening [15].

### 3.2. Conventional Treatment

Management of RA-related dry eye starts with local therapy aimed at improving lubrication and reducing surface inflammation. First-line treatments include artificial tear drops applied frequently (preferably preservative-free when used more than four times daily) to moisten the ocular surface and lubricant ointments for nighttime use [7,16]. Lid hygiene and warm compresses are recommended when meibomian gland dysfunction contributes to evaporative symptoms. Omega-3 polyunsaturated fatty acids may reduce ocular surface inflammation and improve meibomian gland secretion quality in patients with DED [17,18,19]. However, the large, multicenter, double-masked DREAM (Dry Eye Assessment and Management) randomized controlled trial (*n* = 535, 64.5% female) did not demonstrate the superiority of omega-3-rich fish oil supplementation (*n* = 349) over olive oil placebo (*n* = 186) for dry eye symptoms or signs over 12 months [20]. Furthermore, evidence for the use of omega-3 in DED comes predominantly from general dry eye populations, not RA-associated DED. As such, extrapolating these findings to RA patients requires caution.

Environmental modifications (such as humidifiers and draft avoidance) and punctual plugs to reduce tear drainage can also help in aqueous-deficient disease [11,21]. In patients with concomitant allergic symptoms (itching, seasonal flares), topical antihistamine or dual-action antihistamine/mast cell stabilizers can be added [22]. Clinicians should also reassess for MGD and medications that exacerbate dryness. If these methods are insufficient, prescription topical immunomodulators may be indicated. Topical cyclosporine A 0.05% eye drops are widely used for inflammatory dry eye, and their use in the treatment of DED has been approved by the Food and Drug Administration (FDA). They inhibit T-cell activation and increase tear production by decreasing ocular surface inflammation and increasing goblet cell density [11,21]. However, they also commonly cause burning, stinging, and increased tearing [21], and evidence for their use in RA-associated DED is extrapolated from general dry eye populations.

In addition, short courses of topical corticosteroids may provide temporary relief during inflammatory flares. However, they should only be prescribed by ophthalmologists for the minimum time necessary due to risk of infection, increased intraocular pressure (IOP), and cataract formation [21]. IOP should be monitored even with short-term use. Oral secretagogues like pilocarpine or cevimeline, which are cholinergic muscarinic receptor agonists, are sometimes used in Sjögren’s syndrome to stimulate tear and saliva production [23]. However, there is a lack of robust evidence for oral secretagogues in non-Sjogren’s dry eye populations. 

### 3.3. Advances and Emerging Therapies

Several new therapies have expanded options for DED, benefiting RA patients with moderate/severe disease. Lifitegrast ophthalmic solution 5%, an inhibitor of lymphocyte function-associated antigen 1 (LFA-1) and intercellular adhesion molecule 1 (ICAM-1) interaction, was approved as an anti-inflammatory eye drop specifically for dye eye disease [24]. In clinical trials, lifitegrast significantly improved dryness symptoms reported by patients compared to placebo, with effects observable as early as two weeks. However, its impact on corneal staining has been inconsistent across trials [25,26,27]. Furthermore, there are no RA-associated DED data, and evidence is extrapolated from general dry eye populations. Higher-concentration cyclosporine drops (0.09%) have also been approved for DED [28], and autologous serum tears have been used. These drops are made from the patient’s own blood serum, which is rich in growth factors and nutrients and helps heal the ocular surface. While not yet FDA-approved, multiple systematic reviews indicate that autologous serum can provide clinically meaningful short-term improvement in dry eye symptoms compared to artificial tears and may also enhance tear film stability [21]. For persistent symptoms despite topical therapy, additional adjuncts include moisture-retaining eyewear and scleral lenses, which can reduce evaporation and provide a protective fluid reservoir over the cornea. In patients with epithelial breakdown, short-term biologic bandage therapy, such as cryopreserved amniotic membrane, may be considered as an adjunct [22,29,30].

Additional emerging options include pharmacologic neuroactivation with varenicline (nicotinic acetylcholine receptor agonist) nasal spray, which stimulates the trigeminal parasympathetic pathways to increase basal tear production [31]. Perfluorohexyl octane ophthalmic solution, an evaporation-targeted therapy administered four times daily, can be considered when evaporative components are prominent [32,33]. Device-based neurostimulation (such as intranasal stimulation) has also been studied, although availability has changed over time [22].

Janus Kinase (JAK) inhibitors, which are used systemically in RA, are being explored for ocular surface disease. These oral small-molecule drugs block the intracellular JAK-STAT signaling pathway, reducing signaling from multiple inflammatory cytokines [34]. Tofacitinib, a JAK 1/3 inhibitor, has been formulated as a topical eye drop in clinical research. In a Phase 1/2 biomarker substudy enrolling 82 patients with moderate to severe dry eye disease, topical tofacitinib (0.005% once daily and 0.003% twice daily) reduced conjunctival Human Leukocyte Antigen-DR isotype (HLA-DR) expression and tear inflammatory markers after 8 weeks [35]. While not FDA-approved for dry eye, the findings suggest that targeted immunomodulators may play a future role in treating dry eye at the ocular surface level.

Finally, systemic disease control can influence ocular surface outcomes. In a prospective study of RA patients using TNF-α inhibitors (*n* = 16), improvements in conjunctival goblet cell density and tear film metrics were reported, suggesting that systemic inflammatory control may contribute to ocular surface improvements in select patients [13].

In summary, the treatment of RA-associated dry eye has evolved from only symptomatic relief (artificial tears) to include disease-modifying topical therapies (such as cyclosporine and lifitegrast) that address underlying inflammation, leading to more effective management of this common condition. A suggested stepwise management framework for RA- associated DED is summarized in Table 1.

## 4. Scleritis

### 4.1. Overview

Scleritis is one of the most severe ocular complications of RA. It includes inflammation of the sclera—the white outer coat of the eye—and can involve adjacent episcleral and corneal tissues [6,9]. RA is the underlying cause in an estimated 8–15% of all scleritis cases, and approximately 2% of RA patients develop scleritis during their disease course [6]. Among patients with immune-mediated diseases and scleritis, RA is the most commonly associated condition (39%) [36]. RA-associated scleritis is often severe, with patients more commonly presenting with diffuse or necrotizing disease [6]. Necrotizing scleritis is a subtype in which patches of sclera undergo avascular necrosis that can progress to globe perforation [9]. In RA-associated necrotizing scleritis, immune complex deposition in scleral vessels activates complementary pathways (particularly the classical pathway via C1q), generating anaphylatoxins (C3a, C5a) that recruit neutrophils and macrophages [37,38,39,40,41,42]. These cells, together with activated T cells and B cells, release matrix metalloproteinases (MMPs), particularly MMP-1, MMP-2, MMP-3, and MMP-9, which are the principal effectors of stromal collagen degradation [43,44,45]. Also, a form of necrotizing scleritis called scleromalacia perforans can occur in longstanding RA, especially in older women [9], as a result of obliterative endarteritis of scleral vessels. This clinically silent, painless condition is characterized by asymptomatic scleral thinning with a bluish hue of the underlying uvea (the eye’s pigmented layer located just beneath the sclera and cornea) and perforation risk [9].

More typically, scleritis causes severe, dull periocular pain, often radiating to the face and scalp (including brow and jaw) and worse at night [9]. Affected scleral areas are tender to palpation, and the redness does not blanch with topical vasoconstrictors (a key distinguishing feature from episcleritis) [9]. Importantly, vision-threatening complications are well documented. Chronic inflammation may lead to scleral thinning, staphyloma formation, adjacent corneal melt, glaucoma, or even loss of the eye [7].

### 4.2. Conventional Treatment

The management of non-infectious scleritis always requires systemic therapy, as purely topical treatment is insufficient for deep scleral inflammation [46]. First, infectious causes must be ruled out (Herpes Zoster, tuberculosis, etc.) since they require pathogen-specific therapy, and immunosuppression can worsen outcomes. Once RA-associated immune scleritis is confirmed, therapy is stepped according to the severity of the condition.

For mild, non-necrotizing anterior diffuse or nodular scleritis with low-grade inflammation, oral NSAIDs (such as naproxen or indomethacin) are appropriate first-line therapy, providing both analgesic and anti-inflammatory benefits [47]. Successful NSAID response is more likely in idiopathic diffuse or nodular scleritis without ocular complications, whereas RA-associated scleritis tends to be more aggressive and may respond incompletely to NSAIDs alone [6]. Topical corticosteroids may offer symptomatic relief but are generally insufficient as monotherapy. If NSAIDs fail to control inflammation within 1–2 weeks (or earlier in the setting of severe pain, decreased vision, posterior involvement, or emerging complications), treatment should be escalated [48].

For moderate to severe disease, systemic corticosteroids are the commonly used main treatment for acute control with oral prednisone (approximately 1 mg/kg/day), with intravenous pulse methylprednisolone reserved for urgent presentations [6,49]. However, corticosteroids are not a long-term solution due to side effects and the high rate of scleritis relapse during tapering [50]. Therefore, a steroid-sparing immunosuppressant such as a disease-modifying antirheumatic drug (DMARD) is typically introduced early, particularly in recurrent disease or when prednisone cannot be tapered below approximately 10 mg/day without a flare, to achieve durable control and minimize steroid exposure [49,51]. In the context of RA-associated scleritis, this often involves optimizing their existing RA therapy or adding another agent. For example, methotrexate, azathioprine, or mycophenolate mofetil are commonly used conventional DMARDs that can help control long-term ocular inflammation [52]. For necrotizing or posterior scleritis, same-day ophthalmology and rheumatology coordination is warranted, with high-dose systemic corticosteroids and prompt systemic evaluation for underlying vasculitis, and escalation to high-efficacy immunosuppression when needed [46]. While cyclophosphamide has historically been used for necrotizing scleritis due to its effectiveness in halting scleral necrosis, recent evidence suggests that other immunosuppressive strategies, including biologic DMARDs for refractory or steroid-dependent disease, may offer comparable efficacy with an improved safety profile [47,53]. 

## 5. Peripheral Ulcerative Keratitis

### 5.1. Overview

Peripheral ulcerative keratitis refers to a crescent-shaped ulceration and inflammation of the peripheral corneal, typically adjacent to the limbus (transition zone between the cornea and sclera). Hallmark features include epithelial defects, stromal destruction, and an inflammatory corneal infiltrate [54].

PUK is thought to result from immune complex deposition within terminal limbal vessels, triggering immune-mediated vasculitis with inflammatory cell infiltration, complement activation, and release of proteolytic enzymes (including matrix metalloproteinases) that drive peripheral stromal melt. Specifically, immune complex deposition activates complement via the classical pathway, generating anaphylatoxins C3a and C5a that recruit neutrophils and macrophages to the limbal stroma [37,41,55,56]. These cells, together with activated T cells, release MMP-1, MMP-2, MMP-3, and MMP-9, which degrade stromal collagen and mediate corneal thinning [37,57,58,59]. TNF-α amplifies this cycle by upregulating MMP expression and sustaining endothelial activation [55,60]. The peripheral cornea is particularly vulnerable because of its distinct vascular anatomy and proximity to limbal immune surveillance [56,61]. This mechanistic framework—immune complex vasculitis, complement-driven recruitment, and MMP-mediated collagen degradation—underpins the therapeutic rationale for systemic immunosuppression and biologic agents in PUK.

In RA, PUK is a serious ocular manifestation that often signals active systemic vasculitis [7]. Although relatively uncommon (occurring in approximately 1–2% of RA patients), RA is the leading cause of noninfectious PUK (34%) [6,62]. Along with necrotizing scleritis, PUK in RA is considered a marker of severe, uncontrolled disease and may reflect the progression of potentially life-threatening systemic vasculitis [62]. Patients usually present with photophobia, acute eye redness, tearing, and severe eye pain [62]. Without prompt treatment, collagenase and immune-complex-mediated destruction in PUK can lead to rapid corneal thinning, perforation of the eyeball, and vision loss [62].

PUK is associated with excess mortality due to systemic vasculitis. The link between severe corneal involvement and increased mortality in RA patients is well-established, reflecting the connection with life-threatening systemic vasculitis [62,63]. This association indicates that RA-associated PUK is not only an eye disease but a manifestation of life-threatening RA vasculitis, necessitating aggressive immunosuppressive therapy and interdisciplinary care.

### 5.2. Conventional Treatment

Treatment of RA-related PUK requires both local ocular measures to preserve the cornea and, crucially, systemic immunosuppression to control the underlying autoimmune assault.

Regardless of etiology, immediate ocular surface protection is required. This may include discontinuing contact lenses, starting intensive preservative-free lubrication, and using broad-spectrum topical antibiotics for epithelial defects [61,62]. Bandage contact lenses can also promote epithelialization and protect the ocular surface. Adjunctive anti-collagenolytic measures (such as oral doxycycline and topical N-acetylcysteine 10–20%, with vitamin C supplementation) may help slow stromal melt and support corneal healing by inhibiting MMP activity [61]. However, while robust experimental data support these interventions, high-quality RCTs in human corneal ulceration are still needed [64,65,66]. Topical corticosteroids can reduce surface inflammation, but they should only be used after infection has been ruled out. Their use is often minimized or avoided in eyes with extreme thinning because they may potentiate corneal melting [67].

Systemic therapy is determined by the underlying cause. Immune-mediated PUK requires urgent systemic immunosuppression in coordination with rheumatology. In RA-associated PUK, induction typically begins with high-dose systemic corticosteroids (prednisone at 1 mg/kg/day, with IV methylprednisolone pulses for rapidly progressive disease or imminent perforation), but steroids should be paired early with a steroid-sparing immunosuppressant [61,62,68]. In an observational RA cohort of 36 patients, initiating immunosuppressive therapy within the first 4 weeks of PUK onset was a protective factor against recurrence, whereas delays beyond 2 months were associated with a higher risk of recurrence and vision loss [68]. Conventional DMARDs, such as MTX (often first line for RA-associated PUK), mycophenolate mofetil, or azathioprine, are used for steroid-sparing control [7]. Cyclophosphamide is generally reserved for necrotizing PUK, concomitant necrotizing scleritis, or established systemic rheumatoid vasculitis [67,69,70].

Structural compromise of the cornea requires urgent tectonic measures alongside systemic therapy. For severe thinning without frank leak, cyanoacrylate tissue adhesive with a bandage contact lens can temporarily stabilize the globe while systemic therapy takes effect [71,72,73]. A positive Seidel test or frank perforation mandates emergent surgical repair with grafts [74].

## 6. Shared Biologic and Targeted Synthetic Therapies for RA-Associated Scleritis and Peripheral Ulcerative Keratitis

The arrival of biologic agents for RA has significantly impacted the treatment of both scleritis and PUK. However, RA-specific evidence remains limited and largely consists of isolated case reports and small retrospective uncontrolled series. As such, treatment choice is typically individualized based on systemic disease phenotype and comorbidities [6].

Use of biologic agents in necrotizing scleritis and PUK is based on their capacity to interrupt immune-complex-mediated vasculitis and downstream inflammatory cascades that drive scleral and corneal destruction [37].

TNF-α is a central mediator in this cascade: it upregulates MMP expression, sustains neutrophil activation, promotes endothelial activation, and amplifies the cycle of vascular inflammation and tissue destruction [37,55,60,75]. Blocking TNF-α with monoclonal antibodies therefore disrupts multiple nodes in this pathway, providing a mechanistic rationale for anti-TNF therapy in both necrotizing scleritis and PUK.

Among anti-TNF-α agents, monoclonal antibodies (infliximab and adalimumab) and the fusion protein etanercept differ in their mechanisms and clinical performance for ocular inflammation [6]. Infliximab, a chimeric anti-TNF monoclonal antibody, has the most RA-specific supportive data for scleritis and PUK; it binds both soluble and transmembrane TNF and can lyse TNF-expressing cells via complementary and antibody-dependent cytotoxicity [76]. For RA-associated scleritis, infliximab has demonstrated effectiveness in multiple case series. In one series of 10 patients with refractory scleritis (including RA patients), 100% had a favorable clinical response, with 60% achieving remission [77]. For RA-associated PUK, all three patients with progressive PUK refractory to conventional immunosuppression had a marked reduction in inflammation and arrets of corneal thinning after receiving infliximab [78]. Adalimumab, a fully human anti-TNF monoclonal antibody, shares a similar mechanism with infliximab (binding both soluble and transmembrane TNF-α) and has also shown promise in RA-associated ocular inflammation [6,79,80,81]. A multicenter study found no significant difference in efficacy between infliximab and adalimumab for refractory uveitis (including scleritis cases) [82]. In contrast, etanercept—a soluble TNF receptor fusion protein that binds only soluble TNF-α (not transmembrane TNF-α)—appears ineffective for RA scleritis and should be avoided in this context [6,83]. A retrospective analysis (*n* = 22) found infliximab significantly more effective than etanercept for ocular inflammation, with 100% of infliximab-treated patients showing an improvement at 1 year versus 0% with etanercept (*p* < 0.001) [70]. The difference is likely attributable to membrane-bound TNF-α on macrophages acting as a potent trigger for inflammatory pathways in ocular tissues, which etanercept cannot adequately neutralize due to its inability to bind transmembrane TNF-α [84].

Rituximab, by depleting CD20+ B cells, reduces the production of pathogenic autoantibodies (including RF and ACPA) and immune complexes that initiate complement activation, offering a complementary mechanism particularly relevant in RF-positive, vasculitis-associated disease [85,86]. Rituximab has been reported to be effective in very short series and isolated cases of rheumatoid scleritis, severe disease with concomitant peripheral ulcerative keratitis, and, sometimes, after cyclophosphamide failure [87,88].

IL-6 blockade with tocilizumab suppresses acute-phase responses and T helper 17 (Th17) cell differentiation, reducing further cytokine-driven MMP upregulation [6,89]. A retrospective case series of 17 patients with refractory ocular inflammatory disease, including 6 patients with scleritis (not limited to RA), suggests that IL-6 inhibition (tocilizumab) may benefit select patients, but robust RA-specific outcomes remain sparse [6,90,91].

JAK inhibitors (tofacitinib, baricitinib, upadacitinib) represent an emerging option in refractory non-infectious ocular inflammation and occupy a distinct position in treatment [92]. JAK inhibitors act intracellularly to block JAK-STAT signaling downstream of multiple cytokine receptors (including IL-6, IL-7, and interferon gamma), providing broad suppression of the inflammatory milieu [92]. As oral agents, they may be preferred when parenteral biologic therapy is not feasible, in patients with inadequate response to biologics, or when the RA disease profile otherwise warrants their use. A prospective cohort from AIDA network registries suggested that JAK inhibitors may reduce relapse frequency in resistant ocular inflammatory disease, but RA-specific scleritis and PUK data remain limited to small series and case reports, and their use for these indications is off-label [92,93]. Important safety considerations must be highlighted: JAK inhibitors are associated with increased risk of serious infections (including herpes zoster reactivation), thromboembolic events, and major adverse cardiovascular events, particularly in patients over 50 with cardiovascular risk factors, a population well-represented among RA patients [94]. These risks are most clearly established for tofacitinib based on the ORAL Surveillance trial [94]. Baricitinib and upadacitinib carry similar class warnings. Therefore, careful patient selection, baseline screening (including tuberculosis, hepatitis B, and cardiovascular risk assessment), and ongoing monitoring are mandatory before initiating JAK inhibitor therapy for ocular RA [94].

In summary, management of RA-associated scleritis and PUK is increasingly moving from prolonged high-dose corticosteroids and cytotoxic therapy to earlier steroid-sparing immunomodulators, including selected biologic and targeted therapies for refractory disease. The pathophysiological rationale—immune complex vasculitis, complement activation, and MMP-driven tissue destruction—directly informs choice of agent. Nevertheless, the field remains constrained by limited controlled data, and prospective studies are needed to define standardized escalation algorithms and optimal patient selection. A suggested treatment escalation framework for RA-associated scleritis according to disease severity is summarized in Table 2 and a suggested management and escalation framework for RA-associated PUK is summarized in Table 3.

## 7. Conclusions and Future Directions

Ocular involvement in rheumatoid arthritis spans a broad clinical spectrum, from common ocular surface disease to less frequent but vision-threatening inflammatory disorders such as scleritis and peripheral ulcerative keratitis. Severe phenotypes often reflect systemic vasculitic activity and should prompt urgent systemic evaluation and coordinated escalation of immunosuppression.

Despite the limitations of existing evidence, several practical principles emerge. First, phenotype and severity should guide urgency: uncomplicated dry eye is typically managed with topical/local measures, whereas scleritis and PUK frequently indicate vasculitic pathology and warrant prompt systemic evaluation. Second, treatment should be approached stepwise, with early steroid-sparing therapy for recurrent or severe inflammation to minimize corticosteroid-related morbidity, consistent with the suggested management frameworks summarized in Table 1, Table 2 and Table 3. Third, multidisciplinary care is essential; alignment between ophthalmology and rheumatology improves systemic disease control, expedites escalation when needed, and ensures appropriate infection screening prior to biologic therapy.

The expanding rheumatology armamentarium is reshaping ocular RA management. Biologic DMARDs, such as TNF-α inhibitors and rituximab, are increasingly used for refractory scleritis and PUK, often enabling corticosteroid tapering. However, comparative effectiveness data are scarce. Early reports on the use of Janus Kinase inhibitors suggest potential benefit in resistant noninfectious ocular inflammation, but safety considerations (such as infection risk, thromboembolism, and cardiovascular events in select populations) underscore the need for careful patient selection and monitoring.

A central challenge in RA-associated ocular disease is the imbalance between clinical urgency and the limited strength of published evidence. Future work should prioritize prospective comparative studies of biologic versus targeted synthetic agents for vision-threatening ocular RA, identification of biomarkers that predict ocular relapse or systemic vasculitis, and integration of standardized imaging endpoints into trials and registries. Such efforts are needed to refine treatment algorithms, improve risk stratification, and, ultimately, preserve vision while minimizing systemic therapy toxicity.

## Figures and Tables

**Table 1:** Suggested stepwise management framework for RA-associated dry eye disease. Key sources [7,11,16,17,21,22,24,28,29,30,31,35].

Clinical Severity	First-Line Local Measures	Escalation Options	Notes/Patient Selection
Mild (intermittent symptoms; minimal staining)	Artificial tears; nighttime ointment; lid hygiene; avoid drafts/use humidifier	Punctual plugs if aqueous-deficient; topical antihistamine/mast cell stabilizer if allergic component	Reassess for meibomian gland dysfunction and medication contributors
Moderate (persistent discomfort; staining; reduced tear breakup time)	Above + preservative-free tears; warm compresses; omega-3 as tolerated	Topical cyclosporine or lifitegrast; short course of low-potency topical steroid for flares	Evidence for omega-3, topical cyclosporine, and lifitegrast is for general dry eye population; expect delayed onset (weeks) for immunomodulators; monitor IOP with steroids
Severe (epithelial defects; vision fluctuation)	Above + lubricating gel; protective eyewear; consider moisture chamber	Autologous serum tears; scleral lenses	Consider concurrent Sjögren syndrome and systemic disease control
Refractory/complex (mixed aqueous + evaporative; inadequate response)	Optimize adherence and technique; treat blepharitis/MGD	Neurostimulation/pharmacologic neuroactivation; evaporation-targeted therapy	Coordinate with rheumatology for systemic inflammation control

**Table 2:** Suggested treatment escalation framework for RA-associated scleritis. Key sources [6,46,47,48,49,51,52,53,76,77,79,80,81,86,89,90,91,92,93,94].

Severity	Initial Therapy	Steroid-Sparing Strategy	When to Escalate/Refer Urgently
Mild anterior diffuse/nodular; no necrosis	Oral NSAID trial; topical adjuncts for comfort	Start DMARD (methotrexate, mycophenolate) if recurrent or steroid-dependent	Failure of NSAIDs in 1–2 weeks; severe pain; decreased vision
Moderate anterior; significant pain; inflammation	Systemic corticosteroids (oral) for acute control	Add DMARD early to taper steroids	Recurrent flares or inability to taper steroids <10 mg/day prednisone equivalent
Necrotizing or posterior scleritis; vision-threatening	High dose systemic corticosteroids ± IV pulse; urgent imaging and systemic work-up	Cyclophosphamide or high efficacy immunosuppression with rheumatology	Same day ophthalmology/rheumatology
Refractory to DMARDs/steroid dependent	Continue short-term steroids as bridge	Biologic DMARD: TNF-α inhibitor; rituximab as alternative or after TNF failure	Screen for TB/hepatitis; monitor for infection

**Table 3:** Suggested management and escalation framework for RA-associated peripheral ulcerative keratitis. Key sources [6,7,61,62,67,68,69,70,71,72,73,74,78,79,80,81,86,92,93,94].

Clinical Scenario	Immediate Ocular Measures	Systemic Therapy	Surgical/Urgent Indications
Suspected PUK/corneal melt	Stop contact lenses; intensive PF lubrication; topical antibiotic prophylaxis; consider anti-collagenase therapy (oral doxycycline, topical N-acetylcysteine; RCT in human cornea still needed); protect ocular surface	Urgent systemic evaluation for vasculitis; start systemic corticosteroids if infection ruled out	Same-day cornea specialist
Imminent perforation (thinning) without frank leak	Bandage contact lens; tissue adhesive (cyanoacrylate)	Add steroid-sparing immunosuppression (MTX, mycophenolate); cyclophosphamide for severe disease	Progressive thinning despite therapy; adjacent scleritis
Perforation/positive Seidel	Cyanoacrylate glue; multilayer amniotic membrane; shield	High-dose systemic immunosuppression with rheumatology	Urgent tectonic graft (lamellar or penetrating) if large or unstable
Refractory disease or relapse on DMARDs	Continue ocular protection and monitoring	Escalate to biologic DMARD (TNF-α inhibitor or rituximab); consider targeted synthetic agents case by case	Multidisciplinary decision; monitor for infection/thromboembolic complications

## Data Availability

No new data were created or analyzed during this study. Data sharing is not applicable to this article.

## Abbreviations

The following abbreviations are used in this manuscript:

ACPA	Anti-citrullinated protein antibody
AIDA	Autoinflammatory Disease Alliance
CD20	Cluster of differentiation 20
DMARD	Disease-modifying antirheumatic drug(s)
FDA	Food and Drug Administration
HLA-DR	Human leukocyte antigen-DR
ICAM-1	Intercellular adhesion molecule 1
IL-6	Interleukin 6
IOP	Intraocular pressure
IV	Intravenous
JAK	Janus kinase
JAK-STAT	Janus kinase–signal transducer and activator of transcription
KCS	Keratoconjunctivitis sicca
LFA-1	Lymphocyte function-associated antigen 1
MGD	Meibomian gland dysfunction
MTX	Methotrexate
NSAIDs	Nonsteroidal anti-inflammatory drugs
PF	Preservative-free
PUK	Peripheral ulcerative keratitis
RA	Rheumatoid arthritis
DED	Dry eye disease
RF	Rheumatoid factor
TB	Tuberculosis
TNF	Tumor necrosis factor
TNF-α	Tumor necrosis factor alpha
AZA	Azathioprine
CYC	Cyclophosphamide
MMF	Mycophenolate mofetil
RTX	Rituximab
RCTs	Randomized controlled trials
DREAM	Dry Eye Assessment and Management Study
MMP	Matrix metalloproteinase
